# miR-573 regulates cell proliferation and apoptosis by targeting Bax in nucleus pulposus cells

**DOI:** 10.1186/s11658-018-0132-y

**Published:** 2019-03-20

**Authors:** Rui Wang, Boping Wen, Dong Sun

**Affiliations:** 1 Department of Massage and Physiotherapy, Guang Xing Hospital, Zhejiang University of Traditional Chinese Medicine, No. 453, Tiyuchang Road, Xihu District, Hangzhou, Zhejiang 310007 People’s Republic of China; 2 Department of Rehabilitation, Western Theater General Hospital, Chengdu, Sichuan 610011 People’s Republic of China

**Keywords:** miR-573, Bax, Nucleus pulposus cells, Intervertebral disc degeneration

## Abstract

**Background:**

MicroRNA (miRNA) plays a vital role in the pathogenesis of intervertebral disc degeneration (IDD). The expression and potential mechanism of miR-573 in human nucleus pulposus (NP) remains to be elucidated. In this study, we aimed to investigate the role of miR-573 in IDD.

**Methods:**

Quantitative reverse transcription polymerase chain reaction (qRT-PCR) analysis was applied to examine the expression of miR-573 and Bax in idiopathic scoliosis tissues and IDD tissues. Human NP cells were employed for analysis. Moreover, the proliferation and apoptosis of NP cells were detected using MTT and flow cytometry assay respectively. The expression levels of Bcl-2, cleaved caspase-3, cleaved caspase-9, caspase-3 and caspase-9 in degenerative NP cells were measured by Western blotting assay. Furthermore, a luciferase reporter assay was used to verify the relationship between miR-573 and Bax.

**Results:**

The results revealed that the mRNA expression level of miR-573 was down-regulated whereas Bax was up-regulated notably in degenerative NP cells. In addition, overexpression of miR-573 increased cell viability remarkably, coupled with inhibition of cell apoptosis. The expression level of Bcl-2 was increased while cleaved caspase-3 and cleaved caspase-9 expression levels were decreased in miR-573 overexpression NP cells. Additionally, the bioinformatics analysis underscored that Bax was a direct target gene of miR-573.

**Conclusion:**

These results suggest that overexpression of miR-573 inhibited NP cell apoptosis by down-regulating Bax, which proved to be a novel effective strategy for IDD therapies.

## Background

Intervertebral disc (IVD) degeneration (IDD) is considered to be the pathological basis of degenerative spinal diseases, leading to intervertebral disc herniation, spinal canal stenosis and lower back pain (LBP) [1]. IDD is depended on the interaction between genetic and environmental factors, which triggers pathogenic responses in disc cell apoptosis and autophagy [2–4]. Although a variety of factors (age, genetic, smoking, infection, environmental) have been reported to influence its etiology, genetics is the main risk factor for degenerative disc disease, accounting for about 70% [5–7]. However, the underlying molecular mechanism of IDD has not been fully elucidated.

MicroRNAs (miRNAs), a kind of short (20–23 nt) noncoding RNAs, inhibit gene mRNA expression levels by directly binding to the 3′-untranslated region (UTR) of target gene mRNAs [8]. It has been well documented that aberrant miRNAs are related to the occurrence and progression of IDD [9–12]. It has been well documented that miR-96 promoted NP cell proliferation by target connecting with ARID/AKT signaling [13]. Furthermore, miR-494 induced cell apoptosis via directly combining with SOX9 in human degenerative NP cells [14]. Therefore, miRNAs might play an important role in the development and progression of IDD through regulating NP cell proliferation and apoptosis. However, the role of miR-573 in IDD has not been fully elucidated.

As is known to us all, the pro-apoptotic protein Bax and the anti-apoptotic protein Bcl-2 are both members of the Bcl-2 family which regulate the mitochondrial functions during apoptosis [15, 16]. When cells are under the status of stress, changes in the membrane are generated, which leads to the release of apoptosis factors into the cytoplasm, including cytochrome C. Then the increasing cytochrome C activates caspases (caspase-3 and caspase-9), which results in accelerated cell apoptosis [17]. Some studies have suggested that the expression of apoptosis relevant factors including cleaved caspase-3, cleaved caspase-9, Bax and Bcl-2 was changed in NP cells [18, 19]. Therefore, after a search on target gene prediction websites, we discovered that Bax might be one of the underlying target genes of miR-573, and relevant research has not been reported.

In the present study, the results revealed that overexpression of miR-573 inhibited cell apoptosis in human degenerated NP cells. Importantly, the underlying regulatory mechanism is negatively regulated by the target gene Bax. To the best of our knowledge, our findings provide a new therapeutic target for the treatment of IDD.

## Methods

### Tissue samples

We obtained IDD tissues (30 cases) and idiopathic scoliosis (IS) tissues (30 cases) from the Hospital Affiliated to Zhejiang University of Traditional Chinese Medicine (Hangzhou, China). All study procedures were approved by the Research Ethics Committee of the Guang Xing Hospital Affiliated to Zhejiang University of Traditional Chinese Medicine. Informed consent was given by all participants. All tissue samples were collected for analysis on obtaining informed consent from all patients. Both tissues were stored at − 80 °C.

### Human NP cell culture (isolation and primary culture)

The isolation of human primary nucleus pulposus cells was described in the previous study [20]. Firstly, after PBS washing, NP degenerated specimens were diced into small fragments of size 2–3 mm^3^. These fragments were incubated in 0.25% trypsin solution for 25 min, followed by treatment with 0.2% type II collagenase (Invitrogen, Carlsbad, CA) at 37 °C for 12 h in DMEM (Gibco, USA) containing 10% FBS, 100 U/ml penicillin and 100 μg/ml streptomycin (Gibco, USA) at 37 °C in an atmosphere of 5% CO_2_. All experiments in this study used first- or second-generation cells.

### Cell transfection

The NP cells were seeded into 6-well plates at the density of 1 × 10^6^ cells/well. When cell reached 70–80% confluence, cells were transfected with mimic control (miR-con) or miR-573 mimic (GenePharma Co., Ltd., Shanghai, China) at a concentration of 40 nM for transfection using Lipofectamine 2000 (Invitrogen, USA) according to the manufacturers’ instructions. Successful transfection was determined by qRT-PCR. The sequence of miR-573 was 5′-CUGAAGUGAUGUGUAACUGAUCAG-3′, miR-573 mimic was 5′-CUGAAGUGAUGUGUAACUGAUCAG-3′, and mimic control was 5′-UCACAACCUCCUAGAAAGAGUAGA-3′. Then the cells were incubated for 24 h to continue the further analysis.

### Quantitative real-time polymerase chain reaction (qRT-PCR)

Total RNA was isolated using TRIzol Reagent (Invitrogen, USA) in accordance with the protocol of the manufacturer. cDNA was synthesized using the QuantiTect reverse transcription kit (Qiagen). The Hairpin-itTM miR-573 qRT-PCR Primer Set (GenePharma Co., Ltd., Shanghai, China) was used to determine the relative quantity of miR-573, and the mRNA level of miR-573 was normalized to the expression of endogenous U6. Bax was measured by SYBR green qRT-PCR assay (Takara), and GAPDH was detected as an endogenous control. The primers used in our study were as follows: miR-573 forward, 5′-ACACTCCAGCTGGGCTGAAGTGATGTGTAA-3′ and reverse, 5′-TGGTGTCGTGGAGTCG-3′; Bax forward, 5′- CCCGAGAGGTCTTTTTCCGAG-3′ and reverse, 5′- CCAGCCCATGATGGTTCTGAT-3′; U6 forward, 5′- CCCCTGGATCTTATCAGGCTC-3′ and reverse, 5′- GCCATCTCCCCGGACAAAG-3′; GAPDH forward, 5′- GGAGCGAGATCCCTCCAAAAT-3′ and reverse, 5′- GGCTGTTGTCATACTTCTCATGG-3′. Quantitative measurements were determined using the 2^−^-^ΔΔCq^ method [21].

### MTT assay

The MTT assay was applied for assessing cell viability. NP cells were divided into untreated (control), miR-con and miR-573 mimic groups. At 24 h after transfection, 5 × 10^3^ cells/well were seeded into 96-well plates to incubate for 48 h. Then 20 μl of MTT was added into each well. Following incubation at 37 °C for 4 h, 200 μl of DMSO was added to the wells. The absorbance was measured with a plate reader at 490 nm.

### Flow cytometry assay

NP cell apoptosis was performed with the Annexin V-allophycocyanin (APC) apoptosis detection kit (BD Pharmingen, San Diego, CA) according to the manufacturer’s protocol. Cells were collected and suspended with 500 μl of Annexin V binding buffer containing 5 μl of APC-labeled Annexin V at the density of 1 × 10^5^ cells/ml. 5 μl of 7-aminoactinomycin-D (7-AAD) was added subsequently and incubated for 15 min in the dark at room temperature. The samples were detected using a flow cytometer (FACSCalibur, BD Biosciences). FlowJo software (Treestar, San Carlos, CA) was applied for analysis.

### Western blot analysis

NP cells were collected and lysed for 25 min in cold RIPA lysis buffer (Beyotime biotechnology, China). The protein concentration was detected using the BCA protein assay (Nanjing Jiancheng Bioengineering Institute, Nanjing, China). Equal amounts of proteins were separated by 10% SDS-PAGE gels, and then transferred to PVDF membranes. After being blocked with 5% non-fat milk for 2 h at room temperature, these membranes were incubated overnight at 4 °C with primary antibodies. Then the membranes were incubated with the goat anti-rabbit horseradish peroxidase-conjugated IgG secondary antibodies (1:1000; cat. no. A0208; Beyotime Institute of Biotechnology, Haimen, China) at room temperature for 1 h. The protein bands were visualized using an ECL Chemiluminescence kit (Millipore, Billerica, MA, USA). Anti-Bax (1:1000; cat. no. 5023 T), anti-Bcl-2 (1:1000; cat. no. 4223 T), anti-cleaved caspase-3 (1:1000; cat. no. 9661 T), anti-cleaved caspase-9 (1:1000; cat. no. 9509 T), anti-caspase-3 (1:1000; cat. no. 9665 T), anti-caspase-9 (1:1000; cat. no. 9508 T) and anti-GAPDH (1:500; cat. no. 5174S) antibodies were purchased from Cell Signaling Technology (Boston, MA, USA). GAPDH was considered as the internal control.

### Luciferase reporter assay

NP cells were seeded in 96-well plates and incubated for 24 h at 37 °C. miR-573 bind sites Bax 3′-UTR-Luc vector with wild type (WT) or mutant (MUT) was established. Vectors were co-transfected with miR-573 mimics or mimic control using Lipofectamine 2000. After 48 h, NP cells were accessed and examined with a luciferase reporter assay system. The transfection efficiency was corrected by a Renilla luciferase vector.

### Statistical analysis

All experiments were repeated at least three times. Data were presented as the mean ± standard deviation. Statistical analysis was carried out using the SPSS 20.0 software (SPSS, Chicago, IL, USA). Differences in mean values between groups were analyzed using Student’s t test or analysis of variance (ANOVA). *P* < 0.05 was considered to indicate a statistically significant difference.

## Results

### Expression levels of miR-573 and Bax in patients with IDD and idiopathic scoliosis tissues

**Fig. 1:**
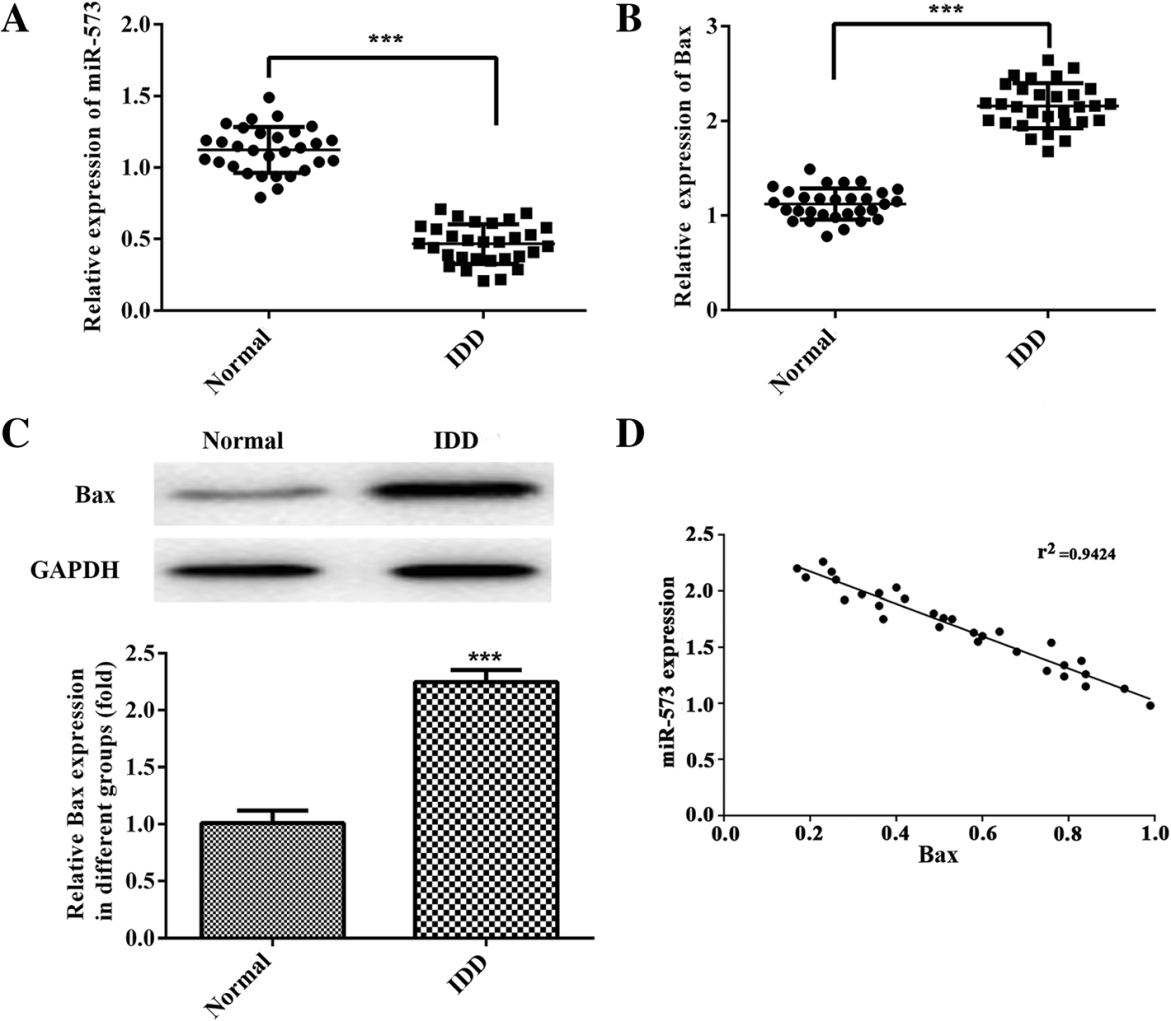
Expression levels of miR-573 and Bax in IS tissues (Normal group) and IDD tissues (IDD group) and the negative correlation between them. Relative expression levels of miR-573 (**a**) and Bax (**b**) were determined by qRT-PCR in human IDD tissues and IS tissues. **c** Protein expression level of Bax in both human IDD tissues and IS tissues. **d** Correlation analysis of mRNA levels between miR-573 and Bax in IDD tissues. ^***^P < 0.001 versus Normal. IS, idiopathic scoliosis; IDD, intervertebral disc degeneration

To explore the role of miR-573 and Bax in IDD, the expression levels of both in IDD and idiopathic scoliosis tissues (Normal group) were evaluated by qRT-PCR assay. As shown in the results, the expression of miR-573 was down-regulated whereas Bax expression was up-regulated in IDD tissues compared with normal idiopathic scoliosis tissues (Fig. 1a and b). Moreover, we found that the protein expression levels of Bax was elevated notably in IDD tissues (Fig. 1c). Importantly, according to the correlation analysis, there was a negative correlation between the mRNA levels of miR-573 and Bax in IDD patients (Fig. 1d, r^2^ = 0.9424). These findings demonstrated that both miR-573 and Bax participate in the development and progression of IDD.

### Overexpression of miR-573 attenuates the expression of Bax in degenerative NP cells

**Fig. 2:**
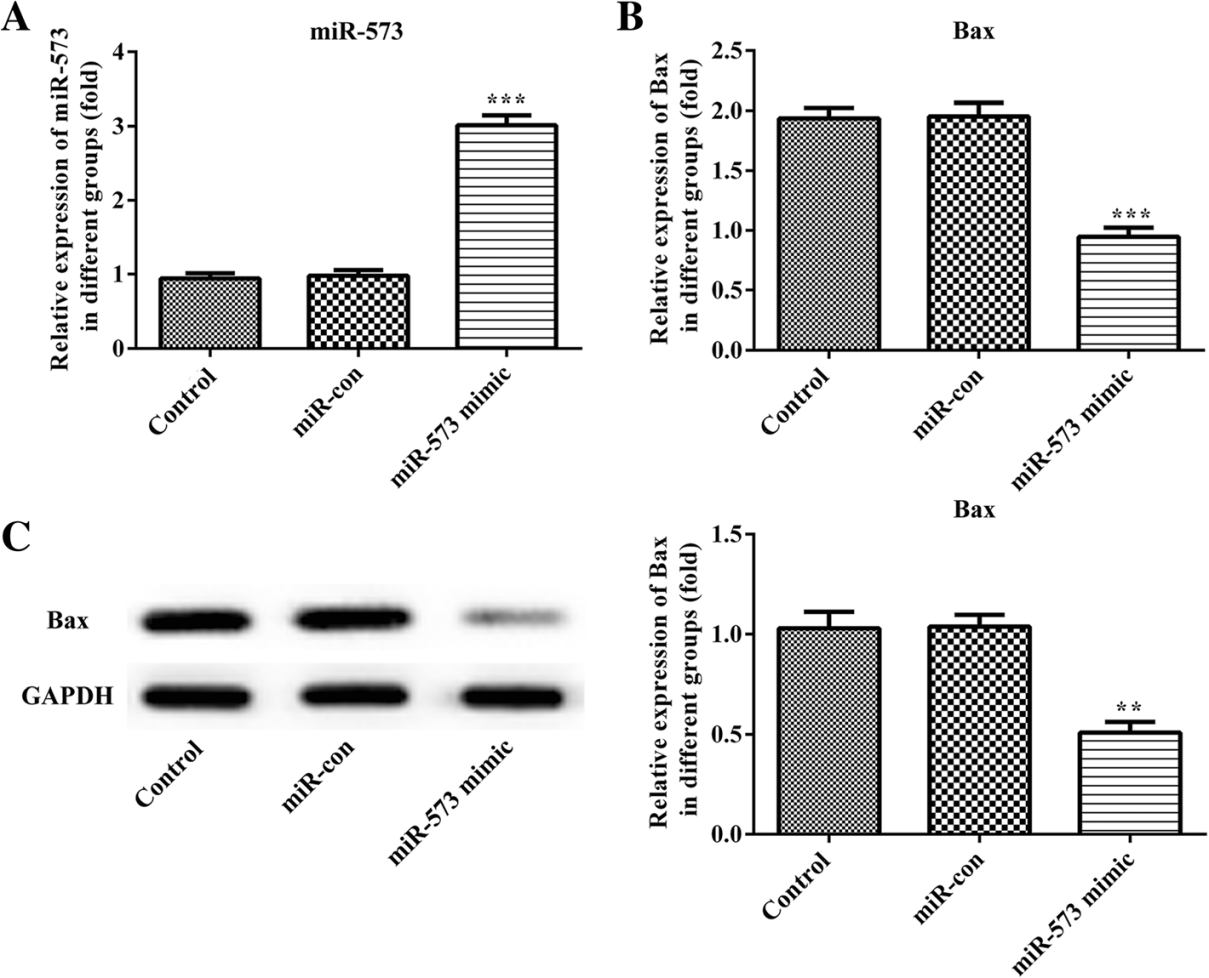
Expression levels of miR-573 and Bax in degenerative NP cells. Cells were transfected with miR-573 mimic, or mimic control for 48 h. Relative expression levels of miR-573 (**a**) and Bax (**b**) were measured by qRT-PCR. **c** Protein expression levels of Bax were measured by Western blot. ^**^P < 0.01, ^***^P < 0.001 versus miR-con

The degenerative NP cells were transfected with miR-573 mimic or mimic control, and the relative expression of miR-573 was verified by qRT-PCR. As shown in Fig. 2a, the miR-573 expression was increased remarkably following overexpression of miR-573 compared with the mimic control group. In addition, the expression of Bax was measured by qRT-PCR and Western blot (Fig. 2b and c). From the results, we found that the mRNA and protein expression levels of Bax were decreased significantly in the miR-573 overexpression group.

### Overexpression of miR-573 increases cell viability and suppresses cell apoptosis in degenerative NP cells

**Fig. 3:**
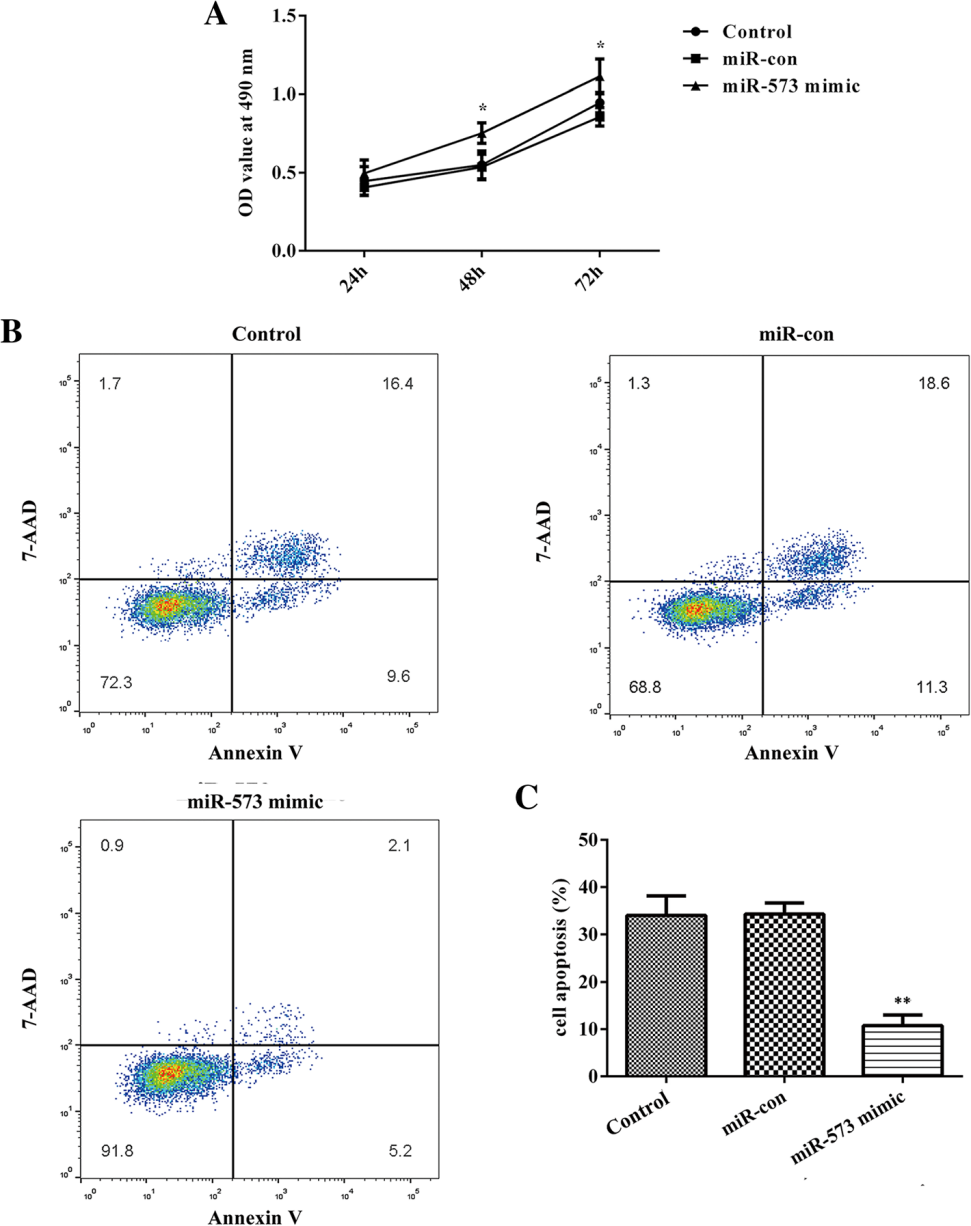
miR-573 modulates cell viability and apoptosis in degenerative NP cells. Cells were transfected with miR-573 mimic, or mimic control for 48 h. **a** Overexpression of miR-573 markedly increased cell viability in degenerative NP cells measured by MTT assay. **b** Overexpression of miR-573 significantly inhibited cell apoptosis in degenerative NP cells by flow cytometry assay. **c** Cell apoptosis was quantified. ^*^P < 0.05, ^**^P < 0.01 versus miR-con

To investigate the roles of miR-573 in cell viability of degenerative NP cells, the MTT assay was employed in our study. As shown in Fig. 3a, overexpression of miR-573 markedly increased NP cell viability. At the same time, cell apoptosis was measured by flow cytometry assay. From the results, we observed that the cell apoptosis level was inhibited significantly after miR-573 overexpression, as exhibited in Fig. 3b and c. Collectively, transfection with miR-573 mimics inhibited cell apoptosis in degenerative NP cells.

### Overexpression of miR-573 increases Bcl-2 expression level accompanied with decreasing levels of cleaved caspase-3 and cleaved caspase-9 expression in degenerative NP cells

**Fig. 4:**
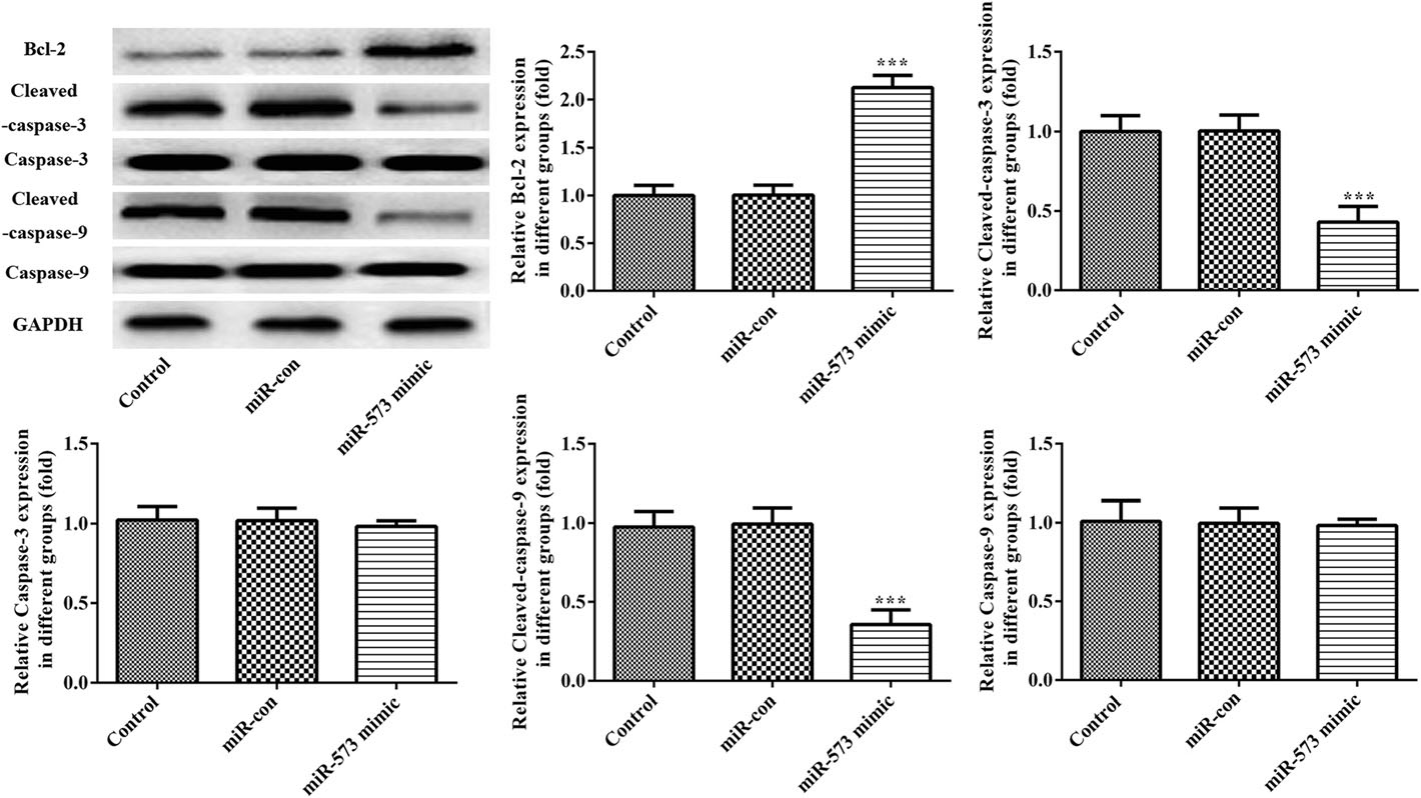
Overexpression of miR-573 markedly increased the expression of Bcl-2 and decreased the expression of cleaved caspase-3 and cleaved caspase-9 in degenerative NP cells. Protein expression levels of Bcl-2, cleaved caspase-3, cleaved caspase-9, caspase-3 and caspase-9 in degenerative NP cells were evaluated using Western blotting assay. ^***^P < 0.001 versus miR-con

As shown in Fig. 4, overexpression of miR-573 remarkably attenuated the expression levels of the anti-apoptotic gene Bcl-2 while inhibiting levels of pro-apoptotic genes cleaved caspase-3 and cleaved caspase-9 in degenerative NP cells. These results indicated that overexpression of miR-573 suppressed cell apoptosis by regulating Bcl-2/caspase-9/caspase-3 pathways.

### Bax is a target gene of miR-573

**Fig. 5:**
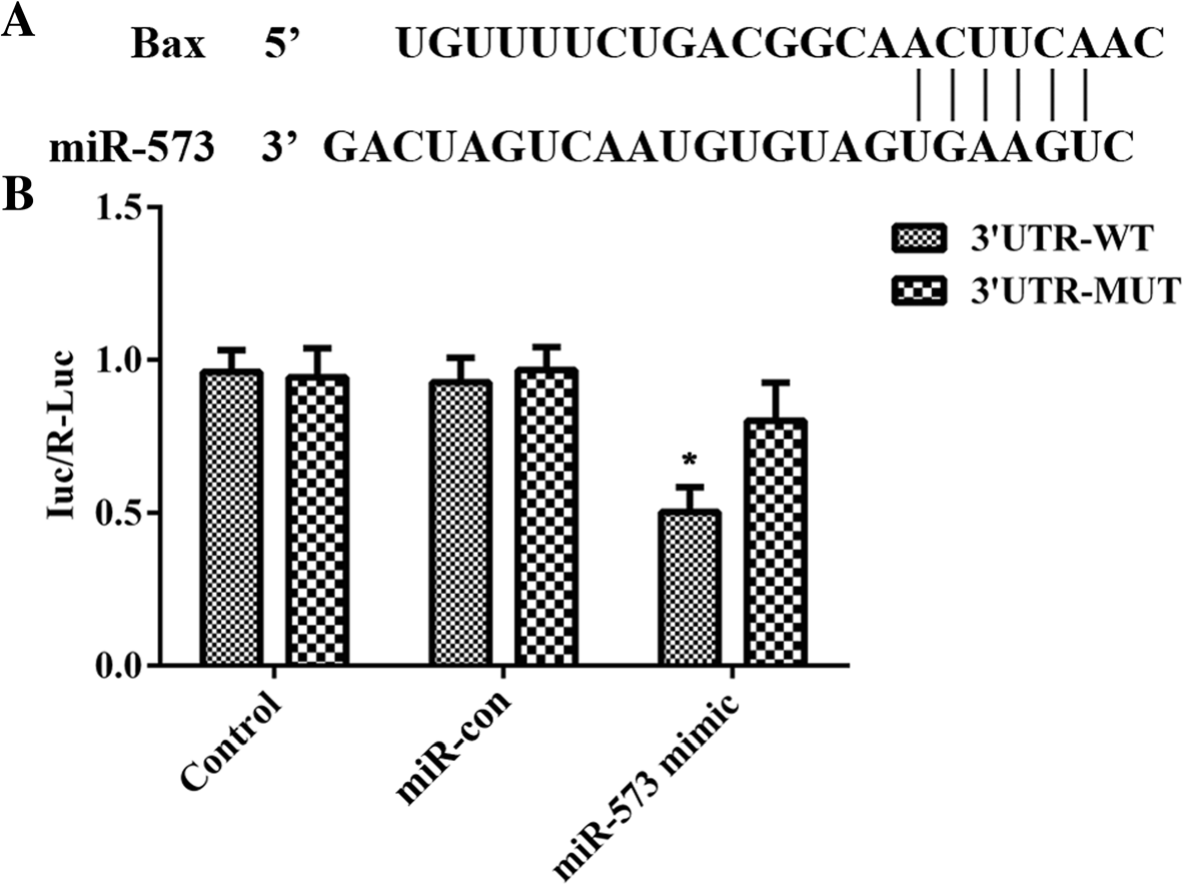
Bax is a target gene of miR-573. **a** Predicted miR-573 target sequence in the 3′-UTR of Bax. **b** Luciferase activity of control, miR-con and miR-573 group in degenerative NP cells. ^*^P < 0.05 versus 3′-UTR-MUT

The TargetScan database was used to verify the potential target mRNA of miR-573 in our study (Fig. 5a). The results suggested that Bax was an assumptive target gene of miR-573. To explore the targeting relationship between miR-573 and Bax, a dual-luciferase activity assay was used in the present study. The results revealed that transfection with miR-573 mimics increased luciferase activity in the MUT 3′-UTR of Bax compared with that of WT (Fig. 5b). To the best of our knowledge, these data indicate that Bax is a direct target gene of miR-573.

## Discussion

Accumulating evidence has suggested that miRNAs play vital roles in diverse pathological and biological processes by impacting on cell proliferation and apoptosis. The functional role of miRNAs in human IDD has been reported as well [22]. For example, miR-494 inhibition protects nucleus pulposus cells by targeting JunD from TNF-α-induced apoptosis [23]. Epigenetic knockdown of miR-143 regulated cell apoptosis in IDD by targeting Bcl-2 [24]. Furthermore, low expression of miR-125a was found in IDD by targeting pro-apoptotic Bcl-2 antagonist killer 1 [25]. However, the roles of miR-573 in the progression of IDD, especially the effects on cell apoptosis and target gene regulation, have not been elucidated yet.

In this study, we found that the expression of miR-573 was decreased in human IDD tissues whereas that of Bax was increased oppositely. More importantly, there was a negative correlation between the mRNA levels of miR-573 and Bax in IDD tissues. Moreover, overexpression of miR-573 decreased the mRNA and protein levels of Bax, which indicated a potential regulatory relationship between miR-573 and Bax. Furthermore, miR-573 overexpression increased cell viability and suppressed cell apoptosis in degenerative NP cells. In addition, there have been no reports regarding the relationship between miR-573 and Bax. Transfection with miR-573 mimics increased luciferase activity in the MUT 3′-UTR of Bax compared with that of WT. Taken together, our present study suggested that miR-573 negatively regulated Bax, which demonstrated that Bax was a direct target of miR-573.

As is well known, apoptosis is a kind of programmed cell death that is stimulated by oxidative stress, DNA damage and inflammatory [26, 27], and it has also been found in IDD [28]. Bcl-2 is an apoptosis inhibitor that plays a critical role in a variety of cell systems through controlling mitochondrial membrane permeability and functions together with caspases and other proteins in a feedback loop system to regulate cell death [29, 30]. Bax is a member of the Bcl-2 family. It has been well documented that increased Bcl-2 expression and decreased Bax expression reduced mitochondrial membrane permeability and suppressed the release of apoptotic activators including cytochrome c, which then attenuates activation of caspase-3 and caspase-9 and leads to inhibition of cell apoptosis [31]. Our results showed that Bax expression was up-regulated in human IDD tissues. After overexpression of miR-573, the protein expression levels of Bcl-2 were elevated while those of cleaved caspase-3 and cleaved caspase-9 were reduced in degenerative NP cells. These results indicated that miR-573 overexpression suppressed cell apoptosis by regulating Bcl-2/caspase-9/caspase-3 pathways.

## Conclusion

Our study revealed that down-regulation of miR-573 and up-regulation of Bax in IDD tissues and miR-573 overexpression increased cell viability and attenuated cell apoptosis of degenerative NP cells via targeting inhibition of Bax. Collectively, these findings indicated that specific regulation of miR-573 might be a novel therapeutic strategy in the development of IDD.

## Abbreviations

IDD: Intervertebral disc degeneration
IS: Idiopathic scoliosis
IVD: Intervertebral disc
LBP: Lower back pain
miR-con: Mimic control
miRNA: microRNA
MUT: Mutant type
NP: Nucleus pulposus
UTR: Untranslated region
WT: Wild type
